# Transcriptome analysis reveals molecular mechanisms responsive to acute cold stress in the tropical stenothermal fish tiger barb (*Puntius tetrazona*)

**DOI:** 10.1186/s12864-020-07139-z

**Published:** 2020-10-23

**Authors:** Lili Liu, Rong Zhang, Xiaowen Wang, Hua Zhu, Zhaohui Tian

**Affiliations:** Beijing Key Laboratory of Fishery Biotechnology, Beijing Fisheries Research Institute, Beijing, 100068 China

**Keywords:** Cold stress, Multiple tissues, Tropical stenothermal fish, Ubiquitin-mediated protein degradation, Heat shock 70 KDa protein, Cold-induced RNA-binding protein

## Abstract

**Background:**

Tropical stenothermal fish exhibit special tolerance and response to cold stress. However current knowledge of the molecular mechanisms response to cold stress in aquatic ectotherms is largely drawn from eurythermal or extreme stenothermal species. The tiger barb *Puntius tetrazona* is a tropical stenothermal fish, with great popularity in aquarium trade and research.

**Results:**

To investigate the response mechanism of *P. tetrazona* to low temperature, fish were exposed to increasing levels of acute cold stress. Histopathological analysis showed that the brain, gill, liver and muscle tissues appeared serious damage after cold stress (13 °C). Brain, gill, liver and muscle tissues from control (CTRL) groups (27 °C) and COLD stress groups (13 °C) of eight-month fish (gender-neutral) were sampled and assessed for transcriptomic profiling by high-throughput sequencing. 83.0 Gb of raw data were generated, filtered and assembled for de novo transcriptome assembly. According to the transcriptome reference, we obtained 392,878 transcripts and 238,878 unigenes, of which 89.29% of the latter were annotated. There were 23,743 differently expressed genes (DEGs) been filtered from four pairs of tissues (brain, gill, liver and muscle) between these cold stress and control groups. These DEGs were mainly involved in circadian entrainment, circadian rhythm, biosynthesis of steroid and fatty acid. There were 64 shared DEGs between the four pairs of groups, and five were related to ubiquitylation/deubiquitylation. Our results suggested that ubiquitin-mediated protein degradation might be necessary for tropical stenothermal fish coping with acute cold stress. Also, the significant cold-induced expression of heat shock 70 kDa protein (*HSP70*) and cold-induced RNA-binding protein (*CIRBP*) was verified. These results suggested that the expression of the molecular chaperones *HSP70* and *CIRBP in P. tetrazona* might play a critical role in coping with acute cold stress.

**Conclusions:**

This is the first transcriptome analysis of *P. tetrazona* using RNA-Seq technology. Novel findings about tropical stenothermal fish under cold stress (such as *HSP70* and *CIRBP* genes) are presented here. This study contributes new insights into the molecular mechanisms of tropical stenothermal species response to acute cold stress*.*

**Supplementary information:**

**Supplementary information** accompanies this paper at 10.1186/s12864-020-07139-z.

## Background

Cold temperature is a major environmental stimulus affecting physiological and metabolic activities, especially for poikilothermic animals [1]. Fish have evolved biochemical and physiological adaptations to the stress of diurnal or seasonal or chronic low temperatures. A conservative responsive mechanism exists in different fish species under long-term low temperature and acute cold stress. For instance, the transcriptional profiling of cold adapting polar fishes implies that routine protein homeostasis is a significant cost in extreme cold and that the ubiquitin-mediated protein degradation pathway is of special importance during this adaptation process [2]. Also, ubiquitination-related genes are significantly expressed in temperate fish such as *Cyprinus carpio* [3–5], tropical fish such as *Danio rerio* [6, 7] and other fishes [8–11] when exposed to acute cold stress.

Temperature tolerance and its underlying mechanisms vary among fish species due to the great variety of ambient temperatures that fish inhabit [8]. The mechanism of temperature stress has been extensively explored in eurythermal and extreme stenothermal fish [2, 5]. Few tropical stenothermal fish species have been reported on with regards to the molecular mechanisms responding to temperature stress. Only damselfish (*Pomacentrus moluccensis*) liver tissue [12] and barramundi (*Lates calcarifer*) muscle tissue [13] have been reported. Genes with functions related to protein turnover, metabolism, and oxidative stress response are identified in damselfish under heat stress [12]. Genes related to the regulation of peptidase activity, microtubule, cytoplasmic and cellular metabolic processes are involved in the responding to heat stress in barramundi muscle tissue [13]. More studies are needed on other species to reveal the response and molecular mechanisms of tropical stenothermal fish after temperature stress [2].

The typical stenothermal tropical fish tiger barb *Puntius tetrazona* (Bleeker, 1855), also named *Systomus tetrazona, Barbus tetrazona* or *Puntigrius tetrazona*, belongs to the family cyprinidae. Tiger barb is a popular aquarium trade fish worldwide [14–18], originating from South-East Asia [14]. In addition, tiger barb is an experimental fish for the study of pathogenic infection [19], parasitic infestations [20], fish diets [17], animal visual perception [21], and the stomach-less teleost digestion system [16]. In countries and regions with wide temperature variation, fish species aquaculture and aquarium distribution logistics is challenging. In cold weather, the tiger barb fish entirely relies on geothermal resources and electrical heating. However, there has been no systematic study of the molecular mechanism underlying cold stress response in tiger barb. A better understanding of acute cold response in stenothermal tropical fish might facilitate the aquarium popularity and experimental use of tiger barb.

Thus, the aims of our study were to 1) evaluate the cold tolerance of the adult tiger barb; 2) identify the genes that were differently expressed under cold stress. Our results have potential applications in the breeding of cold-resistant tiger barb species for aquaculture.

## Results

### Survival analysis and morphological changes of *P. tetrazona*

**Fig. 1:**
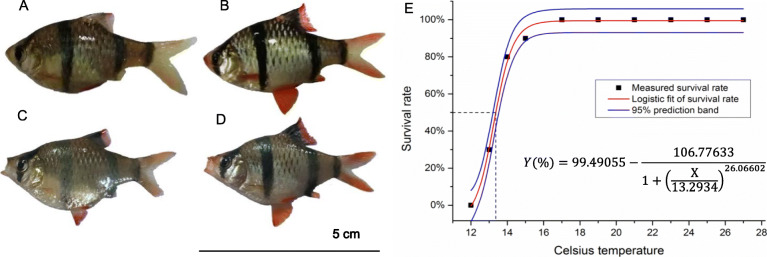
The body color and survival rate of *P. tetrazona* under gradient cold stress. **a** female fish in CTRL group; **b** male fish in CTRL group; **c** female fish in COLD group; **d** male fish in COLD group; **e** the logistic fit curve of survival rate under gradient cold stress. The animal images were taken by Dr. Lili Liu

The tiger barb body color is generally silver to brownish yellow, with four vertical solid black stripes (Fig. 1a). During spawning season, males exhibit bright red (nuptial coloration) in the dorsal fin, ventral fin, caudal fin and snout (Fig. 1b). When the water temperature drops to 21 °C, both male and female fish have been observed to have decayed body color, especially the nuptial coloration in males. When the temperature decreases to 19 °C, the distinctive black stripes even appear dimmed (Fig. 1c, d), in addition to the feeding and locomotion of tiger barb being reduced dramatically. The fish stopped feeding and lost balance completely at 15 °C. The temperature window of tiger barb fish aquaculture is thus 21 °C -27 °C (Fig. 1e). A logistic fit curve and a 95% confidence interval for the survival rate of *P. tetrazona* under a gradient cold stress were thus achieved (Fig. 1e). The celsius temperature was computed as 13.29 ± 0.07 °C when the survival rate was 50% (Fig. 1e). This theoretical value was confirmed by later experiments which showed a 50% survival rate at a measured temperature of 13 °C − 13.5 °C.

**Fig. 2:**
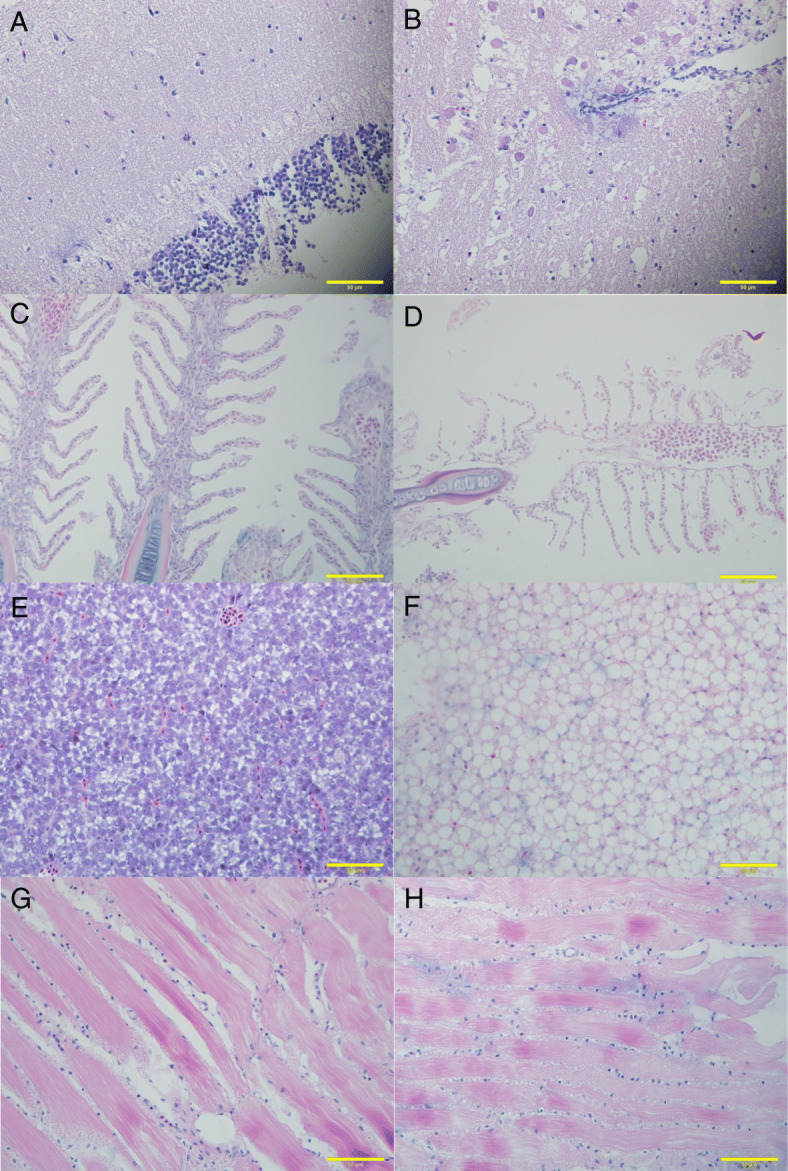
Hematoxylin-eosin staining of paraffin sections of the adult tiger barb from CTRL group (27 °C) and COLD group (13 °C). **a** the brain tissue in CTRL group; **b** the brain tissue in COLD group; **c** the gill tissue in CTRL group; **d** the gill tissue in COLD group; **e** the liver tissue in CTRL group; **f** the liver tissue in COLD group; **g** the muscle tissue in CTRL group; **h** the muscle tissue in COLD group. Scale bar = 50 μm

Hematoxylin-eosin (HE) staining of paraffin slices showed that the brain, gill, liver and muscle tissues suffered serious histopathological damages after cold stress (13 °C). Compared to the brain tissue of a control (CTRL) group (27 °C, Fig. 2a), the brain tissue of the COLD group showed vacuolation and cytocinesis in some neuron cells (Fig. 2b). Compared to the CTRL group (Fig. 2c), the cells in the gill tissue of the COLD group showed shrinkage membrane contraction and even fracture (Fig. 2d). Compared to the CTRL group Liver tissue (Fig. 2e), severe fatty degeneration was induced in the hepatocytes of COLD group fish (Fig. 2f). The myofibers of the CTRL group fish muscle was covered by sarcolemma with well-arranged myofibrils in the sarcoplasm (Fig. 2g), while the myofibrils were disorganized and irregular in the myofibers of COLD group fish muscle (Fig. 2h).

### De novo transcriptome assembly of *P. tetrazona*

Raw data (1,183,577,726 reads) were achieved and submitted to the SRA database (Accession Number: SRP153005). A total of 172.49 Gb of clean data (1,149,935,440 reads) was obtained after removing reads containing adapters or reads of low quality. We then performed de novo transcriptome assembly with clean data using Trinity. The assembly yielded 392,878 transcripts and 238,878 unigenes. The latter included 176,914 non-coding genes and 61,964 coding genes according to the LGC (https://bigd.big.ac.cn/lgc/). A length analysis and distribution were presented in Table 1. The reads quantity and quatity is given in Supplementary Table 1. The mapping rate for each sample is listed in Supplementary Table 2. CEGMA software showed that 241 genes were assembled from 248 core eukaryotic genes (completeness 97.18%) (Table 2), and BUSCO assessment showed that 69.4% complete BUSCOs and 45.1% duplicated BUSCOs for 238,878 uningenes, 89.5% complete BUSCOs and 59.5% duplicated BUSCOs for 61,964 coding genes (Table 2). Some BUSCO proteins corresponded to 2–6 contigs, leading to the high duplicated BUSCO value.

**Table 1:** Summary of Length of transcripts and unigenes

The numbers of transcripts and unigenes for different types of length intervals							
Transcript length interval		200–500 bp	500 bp-1000 bp		1000–2000 bp	> 2000 bp	Total
Number of transcripts		175,952	79,784		58,804	78,338	392,878
Number of unigenes		41,229	60,754		58,561	78,334	238,878
The length distribution of transcripts and unigenes							
	Min Length	Mean Length	Median Length	Max Length	N50	N90	Total Nucleotides
Transcripts	201	1292	580	37,731	2729	462	507,461,187
Unigenes	201	1899	1218	37,731	3107	852	453,596,462

**Table 2:** De novo transcriptome assembly assessment results

Total unigenes	BUSCO	Complete BUSCOs	Fragmented BUSCOs	Missing BUSCOs	Total BUSCO groups searched
		91.9%^a^	7.2%	0.9%	2586
	CEGMA	Complete		Complete+partial	
		Prots^b^	Completeness^c^	Prots^b^	Completeness^c^
		227	91.53	241	97.18
Coding genes	BUSCO	Complete BUSCOs	Fragmented BUSCOs	Missing BUSCOs	Total BUSCO groups searched
		71.9%^d^	7.7%	20.4%	2586

^a^: Complete single-copy BUSCOs occupied 27.4%; Complete duplicated BUSCOs occupied 464.5%

^b^: The number of assembled core genes

^c^: The percent of assembled core genes in core gene set

^d^: Complete single-copy BUSCOs occupied 23.0%; Complete duplicated BUSCOs occupied 48.9%

Three biological replicates were utilized, as is common convention in biological experimentation [22]. In the present study, the squares of the Pearson correlation coefficient (R^2^ values) fore gene expression values between biological replicates were larger than 0.8, indicating the high repeatability of biological replicates. R^2^ values between the COLD and CTRL groups of brain/gill/muscle tissue ranged from 0.7 to 0.8, which suggested that tissues before and after cold treatment shared expression patterns. Meanwhile, the R^2^ values between most different tissues were less than 0.5, which suggested less similarity in the gene expression patterns of multi-tissues (Supplementary Fig. 1).

### Gene annotation and functional classification

**Fig. 3:**
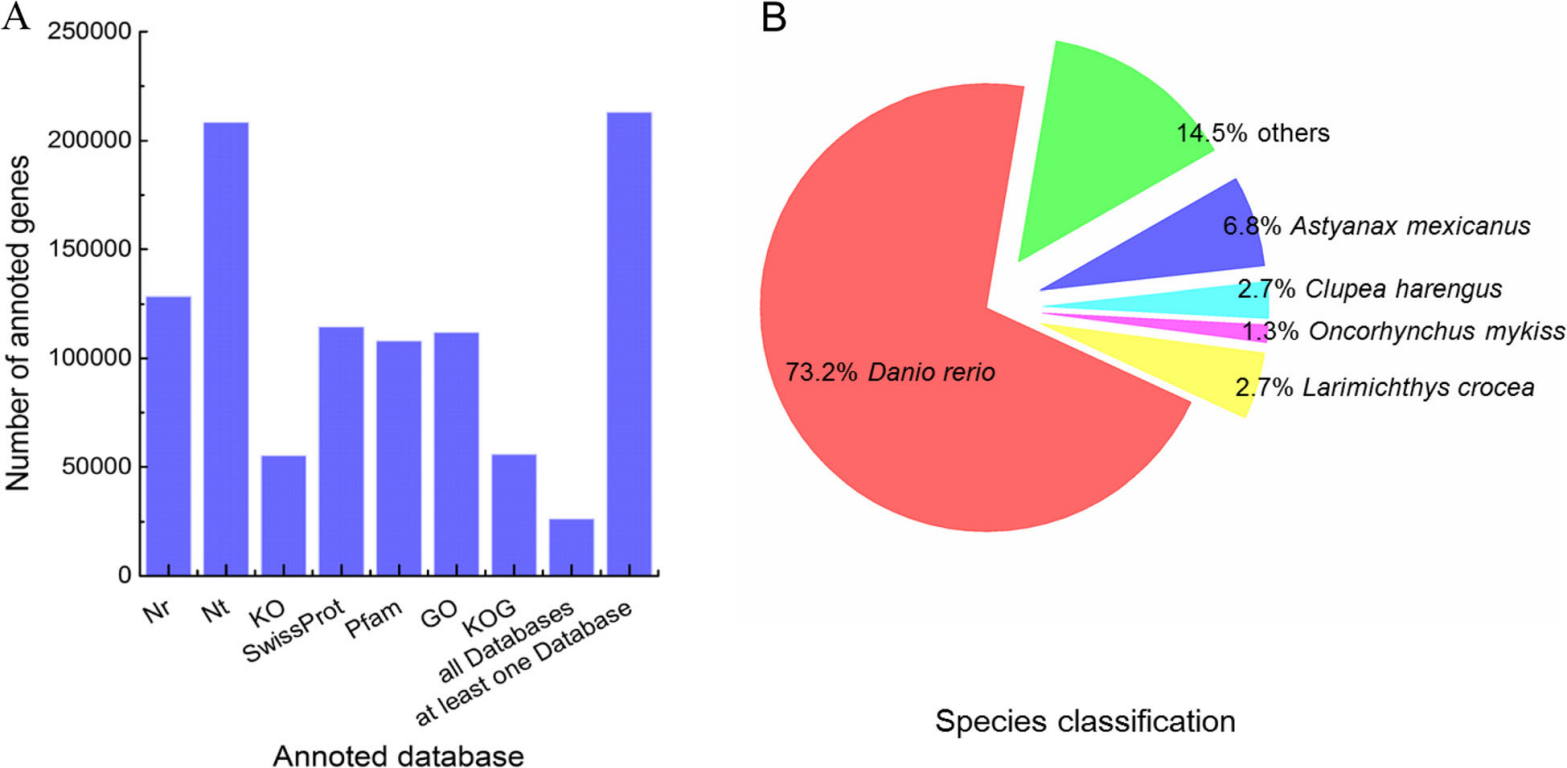
Gene annotation and species classification of *P. tetrazona* transcriptome data. **a** the number analysis of annoted genes in each database; **b** the species classification pie

There were 238,878 unigenes of *P. tetrazona* predicted, and 213,317 unigenes (89.29% of the total) annotated in at least one database from NCBI non-redundant protein sequences (Nr), NCBI nucleotide sequences (Nt), Protein family (Pfam), EuKaryotic Orthologous Groups (KOG), Swiss-Prot, Kyoto Encyclopedia of Genes and Genome (KEGG) and Gene Ontology (GO) (Supplementary Table 3). In particularly, 128,722 unigenes (53.88%) matched to the Nr database, and 114,648 (47.99%) unigenes matched to the SwissProt database with 95,643 unigenes (40.04%) being shared by the two databases. There were 26,398 unigenes (11.05%) beenannotated in all seven databases (Fig. 3a). The function and sequence similarity of annotated unigenes can be predicted based on database annotations.

According to the gene annotation results from the Nr database, the species distribution of annotated unigenes was further analyzed (Fig. 3 b). The vast majority of matched unigenes (73.2% of the total) showed similarity to sequences of from zebrafish (*D. rerio*), followed by the blind cave tetra (*Astyanax mexicanus*) (6.8%), Atlantic herring (*Clupea harengus*) (2.7%), large yellow croaker (*Larimichthys crocea*) (1.5%), rainbow trout (*Oncorhynchus mykiss*) (1.3%) and others (14.5%).

### DEGs in multi-tissues under acute cold stress

**Fig. 4:**
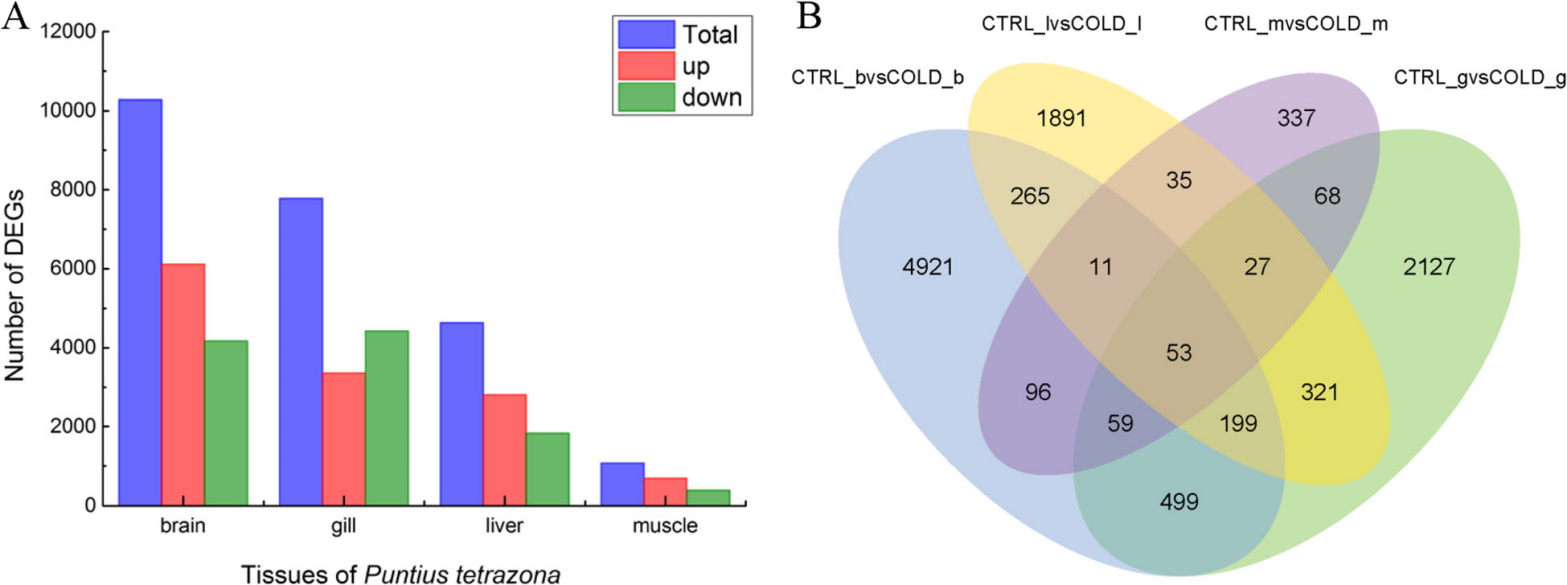
The differently expressed genes (DEGs) analysis. **a** the numbers of DEGs in multi-tissues after cold stress. **b **the venn diagram of DEGs in multi-tissues after cold stress. DEGs between low-temperature groups and control groups of brain, gill, liver and muscle were screened with the threshold *Padj* < 0.05

A total of 23,743 differently expressed genes (DEGs) between low-temperature groups and control groups from multi-tissues (CTRL_b vs COLD_b, CTRL_g vs COLD_g, CTRL_l vs COLD_l, CTRL_m vs COLD_m) were identified (*Padj* < 0.05). The largest number of DEGs was identified in brain tissue. There were 10,272 DEGs, including 4169 up regulated genes (40.6%) and 6103 down regulated genes (59.4%). A total of 7771 DEGs consisting of 4418 up regulated genes (56.9%) and 3353 down regulated genes (43.1%) were verified in the gill tissue. There were 4632 unigenes expressed differently in the liver tissue, including 1830 up regulated genes (39.5% of the total) and 2802 down regulated genes (60.5%). The least number of unigenes were differently expressed in muscle tissue. A total of 1068 DEGs were identified in muscle tissue, consisting of only 382 up-regulated genes (35.8%) and 686 down-regulated genes (64.2%) (Fig. 4a).

Cold-induced DEGs in the brain, gill, liver and muscle shared some common ground (Fig. 4b). The number of genes that were collectively expressed within each group was 64, among which were mainly genes encoding GTPase, ubiquitin ligase/ hydrolase, signaling receptor protein and RNA/DNA binding proteins. Among these 64 genes, five genes involved in ubiquitylation/deubiquitylation.

According to Gene Ontology (GO) analysis, the DEGs from CTRL_g vs COLD_g were enriched mainly in binding (4250 genes), protein binding (2383 genes), immune system process (191 genes), immune response (146 genes) and monocarboxylic acid metabolic process (166 genes). In liver tissue, DEGs were classified into 42 GO terms, and the most enriched terms were metabolic process (2242 genes), catalytic activity (1932 genes), single-organism metabolic process (1152 genes) and small molecule metabolic process (673 genes). DEGs from CTRL_b vs COLD_b were highly enriched in binding, protein binding and circadian regulation of gene expression. DEGs from CTRL_m vs COLD_m were sparsely classified into many different terms, especially biological processes such as heterocycle biosynthetic process (197 genes), aromatic compound biosynthetic process (196 genes), nucleobase-containing compound biosynthetic process (179 genes), cellular localization (101 genes), establishment of localization in cell (95 genes), intracellular transport (88 genes), cellular protein localization (73 genes), cellular macromolecule localization (73 genes) and intracellular protein transport process (67 genes) (Supplementary Fig. 2).

**Fig. 5:**
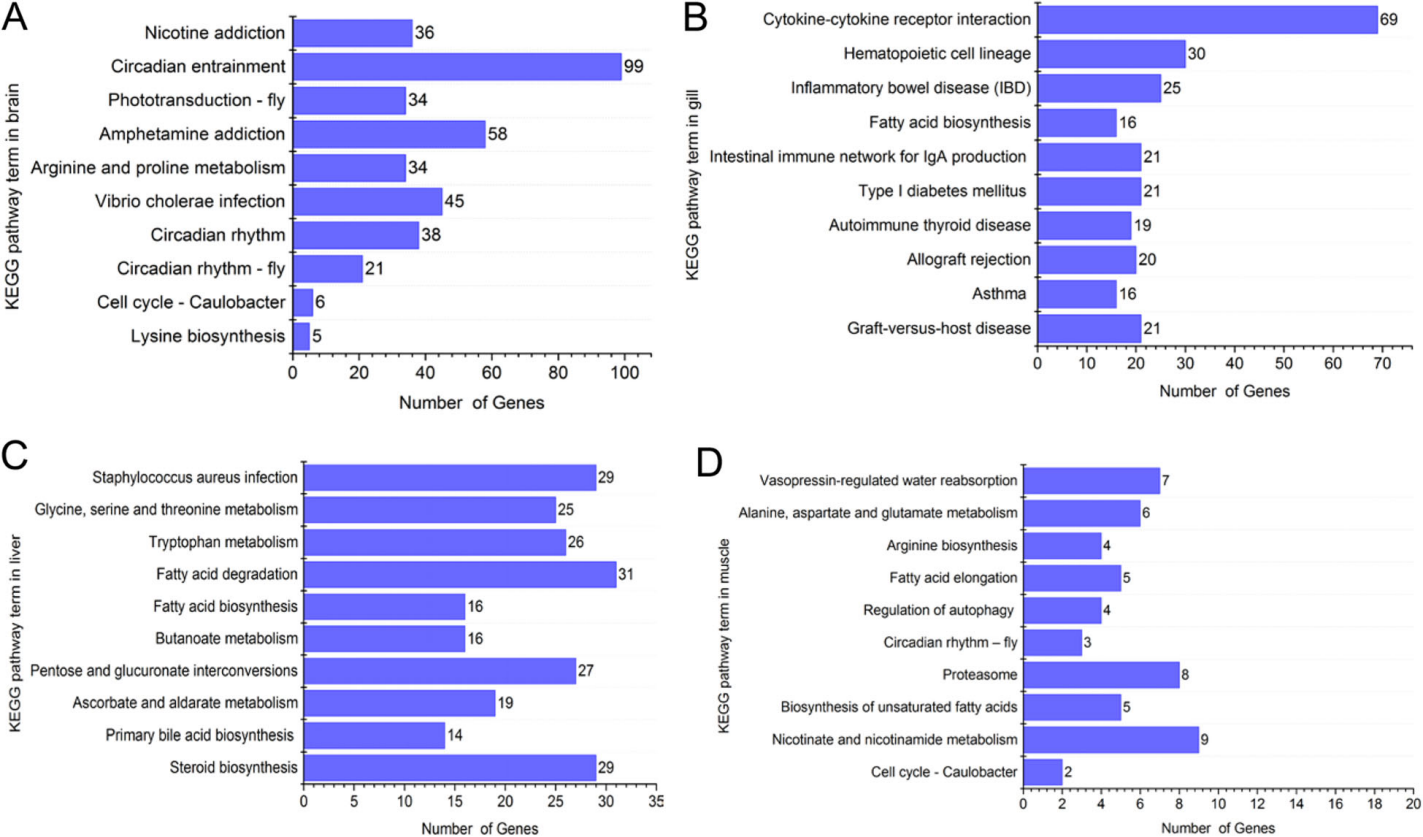
Top10 Kyoto Encyclopedia of Genes and Genomes (KEGG) enrichment pathways of the differently expressed genes. Acute cold-induced DEGs from brain (**a**), gill (**b**), liver (**c**) and muscle (**d**) tissues were enriched in KEGG pathway

The top 10 significantly enriched KEGG pathways (based on *Padj* value) in each tissue were reported in the present study. In brain tissue, many DEGs were involved in circadian rhythm. There were 99, 38 and 21 DEGs enriched respectively in circadian entrainment, circadian rhythm and circadian rhythm-fly pathways (*Padj* < 0.05) (Fig. 5a). Cold-induced DEGs in gill were mostly enriched in the immune system. There were 25, 21 and 19 DEGs enriched in inflammatory bowel disease, intestinal immune network for immunoglobulin A production and autoimmune thyroid disease pathways, respectively (*Padj* < 0.05). Other KEGG pathways enriched in gill DEGs included asthma, type I diabetes mellitus and fatty acid biosynthesis (Fig. 5b).

In liver tissue, cold-induced DEGs were classified mainly into metabolism and biosynthesis pathways (*Padj* < 0.01), including steroid biosynthesis (29 genes), primary bile acid biosynthesis (14 genes), fatty acid biosynthesis (16 genes), ascorbate and aldarate metabolism (19 genes), butanoate metabolism (16 genes), tryptophan metabolism (26 genes), glycine, serine and threonine metabolism (25 genes), pentose and glucuronate interconversions (27 genes) and fatty acid degradation (31 genes) (Fig. 5c). In muscle tissue, DEGs were poorly concentrated, even dispersed (Fig. 5d).

### Relative quantification of ubiquitylation/deubiquitylation-related genes in multi-tissues under gradient cold temperatures

**Fig. 6:**
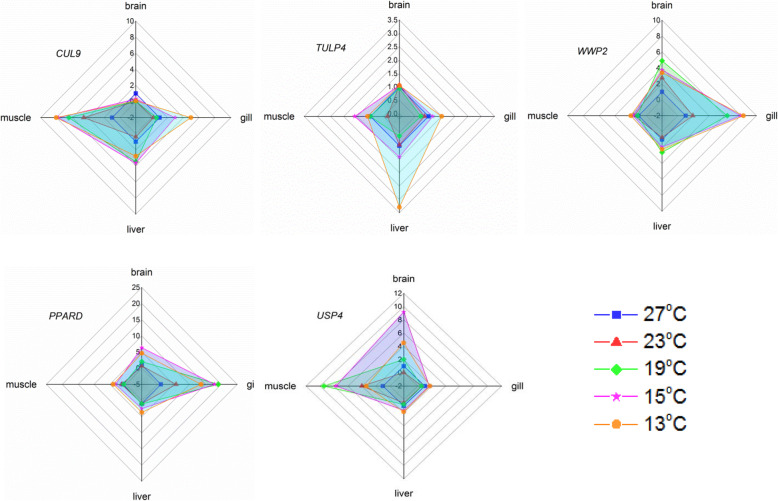
Radar analysis of the temperature-dependent tissue-specific expression of ubiquitylation/deubiquitylation-related genes. Five ubiquitylation/deubiquitylation-related genes *CUL9, TULP4, WWP2, PPARD* and *USP4* in brain, gill, liver and muscle were further analyzed after gradient cold temperature stress (27, 23, 19, 15 and 13 °C)

Five ubiquitylation/deubiquitylation-related genes were identified as cold-induced DEGs in all brain, gill, liver and muscle tissues tested. They were annotated as tubby-related protein 4 isoform X1 (TULP4), NEDD4-like E3 ubiquitin-protein ligase (WWP2), peroxisome proliferator-activated receptor delta (PPARD), cullin-9-like isoform X3 (CUL9) and ubiquitin carboxyl-terminal hydrolase 4 (USP4), according to the Nr database. The differential expression of these five ubiquitylation/deubiquitylation-related genes was then confirmed by qPCR. Melt curve analysis revealed single curves for all primers, suggesting the tested primers were specific and the assembled transcriptome sequence was reliable. qPCR results suggested these five ubiquitylation/deubiquitylation -related genes were induced by acute low temperatures in a tissue specific manner (Fig. 6).

The expression levels of *CUL9* increased under different cold temperatures in the gill, liver and particularly in muscle (up to 8-fold change relative to the control group). The expression of *TULP4* was induced significantly in the liver, followed by the gill and muscle under acute cold stress. The expression of *WWP2* was more sensitive to acute cold stress in the gill rather than in the brain or the liver. The expression levels of *PPARD* increased in multi-tissues after acute cold stress, with the fold change rank from high to low being gill> brain>liver>muscle. The acute cold-induced expression of *USP4* was found mainly in the muscle and the brain (Fig. 6).

### Cold-induced expression of HSP70 and CRIBP in multi-tissues

**Fig. 7:**
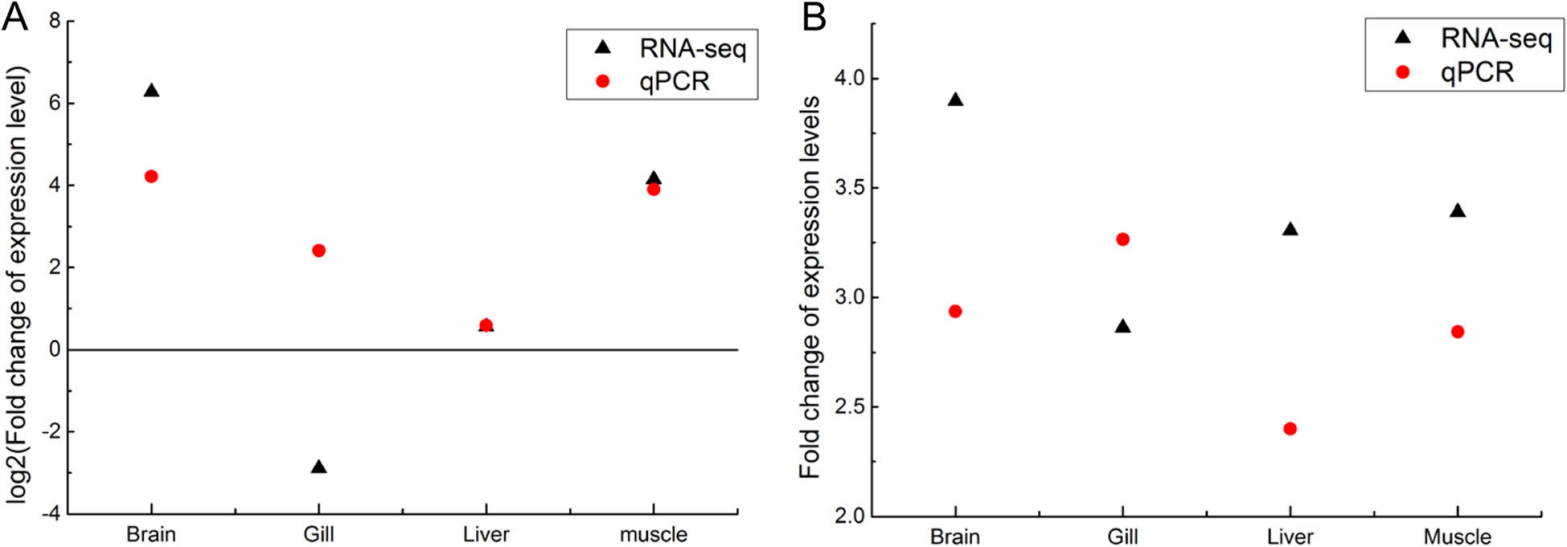
The fold change analysis of cold-induced expression of *HSP70* and *CRIBP* in *P. tetrazona* multi-tissues based on RNA-seq and qPCR methods. **a** the fold change analysis of *HSP70* in COLD groups compared to CTRL groups. **b** the fold change analysis of *CRIBP* in COLD groups compared to CTRL groups. The black letter a indicates *Pad*j < 0.05 for RNA-seq results. The red letter b indicates *P* < 0.01 for qPCR results

According to our RNA-seq data, the gene encoding heat shock 70 kDa protein (*HSP70*) was differently expressed in the brain (77.7-fold) and the muscle (16.7-fold) after acute 13 °C cold stress (*Padj* < 0.05). Our qPCR results confirmed this significant difference in the brain and the muscle with a up-regulation of 18.6 and 15.0 fold (*P* < 0.01). Both the RNA-seq and qPCR data showed a weak increased expression in the liver after cold stress. However, the opposite expression trend was found in gill tissue using two methods, and one possible reason might be the low absolute expression levels in the gill (Fig. 7a).

Though only the expression of the gene encoding cold inducible RNA binding protein coding gene (*CRIBP*) in the brain (3.9-fold) and gill (2.9-fold) tissues was significantly induced by acute cold stress (13 °C) according to our RNA-seq data (*Padj* < 0.05), the expression levels of *CRIBP* were up regulated significantly in the brain, gill, liver and muscle tissues with 2.9, 2.3, 2.4 and 2.8 fold after acute cold stress based on qPCR results (*P* < 0.01) (Fig. 7b).

## Discussion

The optimum growth temperature for tropical fish is usually above 20 °C and particularly sensitive to low temperatures. The window temperature for *P. tetrazona* is 21-28 °C, indicating the tiger barb a typical stenothermal tropical fish. Cold stress is indicated by the decayed nuptial coloration in *P. terazona*. The male nuptial coloration represents his social hierarchy and health, influencing mating choices [23]. Decayed nuptial coloration under mild cold stress (21 °C) indicates adverse effects on health conditions and mating choice. Muscle [24], gill [25], liver and brain [26] are the main target organs of cold stress in fish. The cell shape and structure organization in these tissues of *P. tetrazona* suffer serious damage under severe cold stress (13 °C).

The present study generated the de novo transcriptome assembly of the stenothermal tropical tiger barb *P. tetrazona* for the first time. A total of 172.49 Gb clean data and 238,878 unigenes were obtained from 24 samples of brain, gill, liver and muscle tissues. The number of unigenes was much higher than genes in most fish species. Similar concern was reported in endemic Schizothoracinae fish [27] and *Aristichthys nobilis* [28] after de novo transcriptome assembly. But a total of 47,394 and 68,431 genes were annotated separately in *D. rerio* (GRCz11) and common carp (*Cyprynius carpio*) according to the NCBI Genome database (https://www.ncbi.nlm.nih.gov/genome, April 29, 2020). Our results indicated that the assembled uningenes contained a large number of non-coding genes (74.1%). The non-coding RNA with poly(A) were not filtered before the library construction. These non-coding genes were involved in the generating of de novo transcriptome assembly.. A The total unigenes and coding genes were separately assessed by BUSCO, revealing high D values. The group of contigs sequences responding to the same BUSCO protein shared similar length but with varied alignment score values. These contigs were often annotated as different genes. A reference transcriptome assembly deserves further study.

The transcriptional profiling of brain, gill, liver and muscle from *P. tetrazona* revealed tissue-specific gene expression patterns and pathways in tiger barb responding to acute cold stress. The stenothermal tropical tiger barb shared some conserved cold-stress responsive mechanisms with extreme stenothermal fish, eurythermal fish and other vertebrates. Our study showed that ubiquitylation/ deubiquitylation-related genes were up-regulated after acute cold stress, giving a hint that ubiquitin-mediated protein degradation was necessary for tropical stenothermal fish coping with acute cold stress. Ubiquitylation is an enzymatic process that involves the bonding of an ubiquitin protein to a substrate protein. This process can lead to the protein degradation, as the substrate usually becomes inactivated and is tagged for degradation by the proteasome through the attachment of the ubiquitin molecule [29].

Ubiquitylation processes have been reported in poikilothermal fish under low temperature stress. Ubiquitin-mediated protein degradation is of special importance for extreme stenothermal fish, such as *Notothenia corriceps*, *Chaenocephalus aceratus* and *Pleuragramma antarcticum* [30]*.* Genes encoding proteins involved in protein biosynthesis and folding are over expressed in extreme stenothermal fish compared to eurythermal fish for the adaption to chronic cold stress. High protein consumption results in enriched transcripts of genes involved in the ubiquitin-mediated protein degradation pathway. A transcriptome analysis of the Antarctic toothfish, *Dissostichus mawsoni* [31] showed that genes encoding proteins related to protein biosynthesis, folding and degradation are up-regulated in the cold-adapted transcriptome when compared with the tropical species zebrafish *D. rerio*. Transcriptomic profiling of brain and liver from three other Antarctic fish, *N. corriceps*, *C. aceratus* and *P. antarcticum* [30] further revealed that this cold-adaptive protein degradation was mediated through ubiquitylation. Consistent with these previous reports, as study suggested that ubiquitylation-dependent protein degradation was a conserved response mechanism for stenothermal and eurythermal fish coping with low temperatures.

In this study, the fatty acid related pathways were involved in different tissues in *P. tetrazona* under low temperature conditions. Transcriptional profiling revealed that steroid biosynthesis, fatty acid biosynthesis and degradation in liver, fatty acids elongation and biosynthesis of unsaturated fatty acids in muscle and fatty acid biosynthesis in the gill were all enriched after cold stress. The sensitivity of unsaturated and saturated fatty acids metabolism in fish under low temperature stress has been reported in many studies. The ratio of unsaturated fatty acid/saturated fatty acid is significantly increased, and the level of cholesterol decreased in studies of various fish, such as Nile tilapia (*Oreochromis niloticus*) [10], milkfish (*Chanos chanos*) and grass carp (*Ctenopharyngodon idella*) [32], gilthead sea bream (*Sparus aurata*) (Linneus 1758) [33], yellow croaker (*Larimichthy crocea*) [9] under low temperature stress. This study suggested that saturated/unsaturated fatty acids were involved in the response to acute cold stress in *P. tetrazona,* and might play key roles in cold resistance [34, 35].

Different expression gene enrichment in *P. tetrazona* after low temperature stress were classified into amino acid metabolism and biosynthesis, including lysine biosynthesis, arginine and proline metabolism in the brain; tryptophan metabolism, glycine, serine and threonine metabolism in the liver; arginine biosynthesis, alanine, aspartate and glutamate metabolism in the muscle. Amino acids are the pool for protein synthesis and functioning, and our results suggested the influence of low temperatures on protein synthesis and function in fish. Our results were consistent with observations in the common carp, whose gene expression in the glycolysis pathway increased under cold stress [5]. The metabolism of amino acids and carbohydrates in organisms is self-regulated to adjust to environmental stressors. Temperatures, in return, affect the metabolism of specific amino acids [36]. The amino acid metabolism and glycometabolism in different fish tissues under cold stress have been reported, including in *Larimichthy crocea* [9], *O. niloticus* [37] and *S. aurata L.* [38], suggesting that this metabolism adjusting mechanism for amino acids and carbohydrates was conserved between tiger barb and eurythermal fish.

This study also showed that DEGs in the brain were classified into circadian regulation and circadian rhythm terms. Research on the relationship between temperature and fish circadian rhythm is relatively scare. Circadian systems regulate essential cellular and physiological processes in organisms. When these systems are perturbed, pathological consequences ensue and disease risk rises [39]. Temperature could act as circadian clock trigger and synchronization factor [40]. Moreover, circadian rhythm can be affected by slight temperature fluctuation little as 2 °C [41]. Transcriptional profiling of brain tissue revealed that the abundance of mRNA related to circadian regulation of gene expression might be driven by cold stress in *P. tetrazona*.

Our study also indicted the expression of the *CIRBP* gene in multi-tissues of *P. tetrazona* and the up regulation under acute cold stress. CIRBP (or A18 hnRNP) belongs to the family of cold shock proteins responding to mild cold shock [42]. CIRBP has been reported in different species ranging from fish [43–45], amphibians [46], mammals [47] and humans [42, 48], with highly conserved functions in cellular processes such as cell survival [49], proliferation [50], circadian modulation [47, 51], telomere maintenance [52] and genome stability [53]. Our data indicated a common molecular response to cold stress in tropical stenothermal fish and other vertebrates.

Additonally, the transcriptomic analysis in the present study revealed a specific cold-stress responsive mechanism in stenothermal tropical fish. Our study validated the inducible expression of *HSP70* in the brain and muscle tissues of *P. tetrazona* under acute cold stress (13 °C), refreshing the perception of *HSP* genes expression in tropical stenothermal fish coping with cold stress [2, 54]. The HSP70 protein family plays a key role in assisting protein folding, repair and degradation as molecular chaperone [55–57]. Both constitutive and inducible HSP70 proteins are widely common in most eurythermal fish such as common carp (*C. carpio*) [58], zebrafish (*D. rerio*) [59], rohu (*Labeo rohita*) [60] and tilapia (*O. niloticus*) [61].

The expression of *HSP70* in stenothermal fish is very complicated. A high abundance of constitutive HSP70 in extreme stenothermal fish is a result of long-term adaption to cold environment [2, 30, 62]. Meanwhile, the loss of inducible HSP70 expression is common with no detection [54] or decreased expression of HSP70 [63] after acute heat shock, demonstrating the loss of heat shock response in extreme stenothermal fish. No induction of the expression of HSP has been reported for now in tropical stenothermal fish under thermal stress, thus a popular view is that the *HSP* genes are just constitutively expressed in tropical stenothermal fish in response to long-term heat stress [2, 12]. Our study supports a different point. The reasons for no observed inducible HSP might come from (a) the species-specific expression caused by an evolutionary difference between fish with dissimilar temperature preferences [64], and tropical stenothermal fish has been under-researched [2]; (b) different molecular mechanism response to cold and heat stress in one same fish species [54, 65]; (c) small sample capacity both for fish species and tissues in previous reports, whichonly included damselfish liver tissue [12] and barramundi muscle tissue [13].

In addition, an increase of ubiquitylation was observed in a tissue-specific manner in multi-tissues of *P. tetrazona* under cold stress. Considering that different E3 ligase can bind with different substrate, our data suggested that different protein-ubiquitylation/deubiquitylation pathways with tissue-specific ubiquitin ligases and substrates functioned in *P. tetrazona* coping with acute cold stress.

The process of ubiquitylation is regulated by three main types of enzymes in entirety, including ubiquitin-activating enzymes (E1), ubiquitin conjugating enzymes (E2) and ubiquitin ligases (E3) [66].TULP4 plays a role as a substrate-recognition component of the SCF-like ECS (Elongin-Cullin-SOCS-box protein) E3 ubiquitin ligase complex which mediates the ubiquitylation and subsequent proteasomal degradation of target proteins. WWP2 serves as an E3 ubiquitin-protein ligase, accepting ubiquitin from an E2 ubiquitin-conjugating enzyme and directly transferring it to targeted substrates, promoting proteasomal degradation [67]. CUL9 is a core component of a Cul9-RING ubiquitin-protein ligase complex, which mediates ubiquitylation and subsequent degradation of BIRC5 (baculoviral IAP repeat-containing protein 5), a key connector for the cell cycle and the apoptosis pathways [68]. PPARD, also named PPAR β/δ, belongs to the family of nuclear receptors that function as transcriptional factors. PPARD regulates ubiquitin C expression and the ubiquitination of proteins is influenced by PPARD [69]. Ubiquitin C (also named polyubiquitin-C) is one of the sources of ubiquitin [70]. USP4 is a ubiquitin specific peptidase 4, belonging to the deubiquitinating enzyme family. USP4 is important in regulating cellular pathways by removing ubiquitin from RIP1 (Rieske iron-sulfur protein), PDK-1 (phosphoinositide-dependent protein kinase-1) and RO52 (52 kDa Ro protein), as well as interacting with SART3 (squamous cell carcinoma antigen recognized by T-cells 3) at the spliceosome [71, 72]. Our study suggested that ubiquitylation/deubiquitylation was prevalently-induced in tiger barb with tissue-specific ligases and substrates after acute cold stress. More details and molecular mechanisms remain unclear and require further investigation.

## Conlusions

The present study was the first report on transcriptional profiling in *P. tetrazona* that we know of. Our data supplements the molecular mechanisms of tropical stenothermal fish responding to low temperature stress, giving a hint to the specific molecular responses to cold stress in tropical stenothermal species compared to extreme stenothermal fish and eurythermal fish. More detailed and comprehensive response mechanisms in multi-tissues from tropical stenothermal fish in response to cold stress deserve further research.

## Methods

### Fish acclimation and acute cold stress

Adult tiger barb (*P. tetrazona*) fish of 8 months were obtained from Zaozhuang aquaculture farm in Shandong Province, China. All fish were fed once a day with commercial dry formula feed. The whole aquaculture system was aerated continuously. After acclimation of 3 weeks in an independent recirculating aquaculture system at temperature 27 ± 0.5 °C, pH 6.5–7.0, kH 6–7. Ninety fish (eight-month old) with similar body length (4.5 cm ± 0.25) were divided into two groups randomly. Forty-five fish in each group were divided equally into four tanks filled with 20 L aquaculture water. One group was the control group (CTRL) in accordance with acclimation conditions and another was the acute cold stress group at decreasing temperature (COLD). The temperature was set to 27 ± 0.5 °C the first day, and descended 2 °C at each of 24 h (_△_T = 2 °C/d) until the temperature reached to 15 ± 0.5 °C, followed by consecutive decreases of △T = 1 °C/d until no survival. The last temperature point was 11–12 ± 0.5 °C. At the end, all fish could not survive. During the process of cold stress, the fish morphology and survival rates were recorded (with the stop of breath as death). The dead fish were removed as soon as discovered. After 24 h at 13 ± 0.5 °C, five fish (gender-neutral) in each tank were anesthetized before opening of the abdominal cavity, dissected by the scissors from the anal region, and the target tissues were weighed up by analytical balance instrument (Melttler Toledo, ME-T). Immediately, biological triplications of brain, gill, liver and muscle tissues from both control (CTRL) group and cold treatment (COLD) group were stored in RNAstore (Tiangen, DP408–02). The water temperature was monitored to keep it at set value±0.5 °C during the whole cold stress experiment.

### Animal euthanasia

The fish were anaesthetised in 90-100 mg/L ethyl 3-aminobenzoate methanesulfonate (Sigma, A5040, MS-222) for 1–2 min before dissection. When the fish were out of balance and unconscious, they were transferred into clean dish and wiped off the solution for dissection.

### Histopathology assay

For hematoxylin eosin (HE) staining, the brain, gill, liver and muscle tissues from CTRL and CLOD groups were fixed with 4% paraformaldehyde (Sigma, 158,127) for 24 h at 4 °C away from light. Then the fixed tissues were trimmed into appropriate size and placed into embedding cassettes for gradient dehydration with ethanol: 60% ethanol for 30 min; 70% ethanol overnight; 80% ethanol for 130 min; 90% ethanol for 130 min, twice; 95% ethanol for 130 min, twice; 100% ethanol for 50 min, twice. The tissues became transparent in new cassettes containing dimethyl benzene for 30 min. The tissues were then transferred into liquid wax at 60 °C for 60 min, twice. The tissues were embedded via histocentre (Kedee, Jinhua China) and taken out when paraffin block containing fish tissues cooled. Tissue cross sections (2–3 μm) were cut with table microtome (Leica RM2016) and mounted onto glass slide. Then rinsed in distilled water for 5 min. Stained in hematoxylin (Sinopharm, Beijing China) solution for 5 min. Rinsed in distilled water for 5 min. Transferred into 1% hydrochloric acid alcohol quickly for 3–5 s. Rinsed in distilled water for 30 s. Transferred into 1% ammonium hydroxide for 10 s. Rinsed in distilled water for 3 min, twice. Then the slices were stained in 0.5% eosin (Sinopharm, Beijing China) solution for 1 min. Rinsed in distilled water for 5 min. Soaked with 80% ethanol until proper differentiation. Transferred into 95% ethanol for 10 s, twice; 100% ethanol for 5 min, twice. Soaked with dimethylbenzene for 5 min, twice. Finally, the slices were mounted in balsam neutral, analyzed by light microscopy (Olympus, BX51) and digital images were taken using the imaging system (Olympus, DP72).

### Total RNA extraction and quality test

Before total RNA extraction, the sample preservation solution should be removed. Added 1 mL TRNzol into the tissue samples (35 ± 5 mg) and homogenized with tissue homogenizer (MP, FastPrep-24). Total RNA from brain, gill, liver and muscle were extracted separately via TRNzol Reagent (Tiangen, DP405) according to the manufacture’s protocol. RNA samples should pass through the checks for RNA degradation, contamination, integrity and quantitation via Nanodrop 2000, agarose gel electrophoresis and Agilent 2100 before library construction. Especially, RNA integrity was assessed using the RNA Nano 6000 Assay Kit of the Agilent Bioanalyzer 2100 system (Agilent Technologies, CA, USA). And the RNA integrity number should be above 6.8.

### Library construction and sequencing

After quality control, mRNA was enriched from total RNA using oligo (dT) beads and then the cDNA library was constructed with NEBNext Ultra II DNA Library Prep Kit for Illumina (New England Biolabs, E7645S) according to the manufacture’s protocol. The final cDNA library concentration was first quantified using a Qubit 2.0 fluorometer (Life Technologies), and then diluted to 1 ng/μL before checking insert size on an Agilent 2100 and quantifying to greater accuracy by quantitative PCR (library activity > 2 nM).

### Assembly and gene annotation

The raw data from Illumina NovaSeq were transformed to sequenced reads by base calling. The raw reads were processed through in-house perl scripts by removing reads containing adapter or ploy-N, and low quality reads to obtain clean reads. Q20, Q30, GC-content and sequence duplication level of the clean data were calculated for quality control. Then the clean reads were assembled with the Trinity software [73] for de novo transcriptome assembly, as no reference genome of tiger barb was released up to now. Hierarchical clustering analysis was carried out with Corset software [74] to remove redundancy, and to separate contigs where existing different expression patterns between samples. The transcripts of a gene might be from alternative splicing, alleles, homologs, orthologs and different copies of a gene according to the Trinity, so the longest transcripts of each cluster were selected as unigenes as officially recommended by Trinity. CEGMA (Core Eukaryotic Genes Mapping Approach, http://korflab.ucdavis.edu/dataseda/cegma/) and BUSCO (Benchmarking Universal Single-Copy Orthologs, http://busco.ezlab.org/) notation assessment were applied for the integrity evaluation of the transcriptome assembly.

To achieve comprehensive gene functional annotation, we performed BLAST analysis against seven databases (Supplementary Table 3), including Nr, Nt, SwissProt, Pfam, GO, KOG and KEGG were applied by the Blast2GO, KAAS, Diamond and NCBI BLAST softwares.

### DEG analysis for GO and KEGG enrichment

De novo transcriptome assembled in the study was used as a reference. The reads were mapped according to reference transcriptome with RSEM [75]. FPKM value was used to account the effects of both sequencing depth and gene length oncounting of fragments. Thus the data was standardized for estimating gene expression levels with FPKM value. For DEG analysis between control and cold stress groups in multi-tissues, the DESeq software [76] was applied based on readcounts (from gene expression level analysis, *Padj* < 0.05). GO enrichment analysis of differently expressed genes (DEGs) was conducted with GOseq [77] based on Wallenius non-central hyper-geometric distribution. KEGG pathway enrichment analysis of DEGs was conducted via KOBAS software [78]. The software information including version and parameters was given in Supplementary Table 4.

### qPCR validation of selected differently expressed genes

For validation, the 1st-strand cDNA of brain, gill, liver and muscle tissue of five fish under 27 °C, 23 °C, 19 °C, 15 °C and 13 °C stress was synthesized via PrimeScript RT reagent Kit with gDNA Eraser (TaKaRa, RR047) based on the manufacture’s protocol. The gene-specific primers of differently expressed genes *HSP70*, *CRIBP*, *TULP4*, *WWP2, PPARD*, *CUL9*, *USP4* and reference gene *βACTIN* were designed according to Primer3web (http://primer3.ut.ee/) (Supplementary Table 5). The *βACTIN* gene sequence identification and stability evaluation was conducted previously (data unpublished). Quantitative real-time PCR (qPCR) was conducted using Talent qPCR PreMix with SYBR GREEN I (Tiangen, FP209) on an ABI FastOnePlus machine (Applied Biosystems). A two-step program method was performed as following: 95 °C 3 min; 40 cycles of 95 °C for 5 s and 60 °C for 30 s. After the program melting curve analysis was done. The data was analyzed using 2^-△△Ct^ relative quantification method [79] with *βACTIN* as inner control. All the experiment was conducted in triplicates. qPCR data was analyzed by StepOne software (version 2.3) with *P* < 0.01. The significance was calculated by one-way ANOVA and post-hoc test with S-N-K and Tukey methods.

## Supplementary information

**Additional file 1.**

## Data Availability

The raw sequences were deposited in the National Center for Biotechnology Information (NCBI) Short Read Archive (SRA) database (http://www.ncbi.nlm.nih.gov/Traces/sra/). The SRA accession number is SRP153005. The other databases information was given in Supplementary Table 3.

## Abbreviations

HE: Hematoxylin-eosin
CEGMA: Core eukaryotic genes mapping approach
BUSCO: Benchmarking universal single-copy orthologs
R^2^: Squares of pearson correlation coefficient
Nr: Non-redundant protein sequences
Nt: Nucleotide sequences
GO: Gene ontology
KO: EuKaryotic orthologous groups
KEGG: Kyoto encyclopedia of genes and genome
DEGs: Differently expressed genes
TULP4: Tubby-related protein 4
WWP2: NEDD4-like E3 ubiquitin-protein ligase
PPAR: Peroxisome proliferator-activated receptor
CUL9: Cullin-9-like isoform X3
USP4: Ubiquitin carboxyl-terminal hydrolase 4
qPCR: Quantitative real-time PCR
HSP70: Heat shock 70 KDa protein
CRIBP: Cold inducible RNA binding protein coding gene
E1: Ubiquitin-activating enzymes
E2: Ubiquitin conjugating enzymes
E3: Ubiquitin ligases
ECS: Elongin-cullin-SOCS-box protein
BIRC5: Baculoviral IAP repeat-containing protein 5
RIP1: Rieske iron-sulfur protein
PDK-1: Phosphoinositide-dependent protein kinase-1
RO52: 52 kDa Ro protein
SART3: Squamous cell carcinoma antigen recognized by T-cells 3
