# Supplementary material for: Transcriptome analysis reveals molecular mechanisms responsive to acute cold stress in the tropical stenothermal fish tiger barb (Puntius tetrazona)
Source: BMC Genomics. 2020 Oct 23;21:737. doi: 10.1186/s12864-020-07139-z (PMC7584086; doi:10.1186/s12864-020-07139-z)
Supplement: Supplementary file 1 — Additional file 1. [file 12864_2020_7139_MOESM1_ESM.docx]

**Supplementary materials**

**Table 1** Reads quantity and quality

| Sample | Raw reads | Clean reads | Clean bases | Error(%) | Q20(%) | Q30(%) | GC content(%) |
| --- | --- | --- | --- | --- | --- | --- | --- |
| CTRL_b_1 | 46,847,258 | 45,451,890 | 6.82 G | 0.03 | 94.73 | 92.52 | 47.12 |
| CTRL_b_2 | 42,854,502 | 41,553,878 | 6.23 G | 0.03 | 95.16 | 93.05 | 46.93 |
| CTRL_b_3 | 51,070,788 | 49,927,048 | 7.49 G | 0.03 | 94.98 | 92.82 | 47.12 |
| CTRL_g_1 | 45,590,250 | 44,181,106 | 6.63 G | 0.03 | 95.23 | 93.14 | 49.23 |
| CTRL_g_2 | 45,188,544 | 44,005,376 | 6.6 G | 0.03 | 94.81 | 92.61 | 48.41 |
| CTRL_g_3 | 57,582,338 | 55,966,124 | 8.39 G | 0.03 | 95 | 92.85 | 48.29 |
| CTRL_l_1 | 57,041,814 | 55,440,394 | 8.32 G | 0.03 | 95.35 | 93.25 | 49.18 |
| CTRL_l_2 | 64,428,992 | 62,428,258 | 9.36 G | 0.03 | 95.71 | 93.7 | 49.12 |
| CTRL_l_3 | 55,856,380 | 54,116,398 | 8.12 G | 0.03 | 95.64 | 93.62 | 48.92 |
| CTRL_m_1 | 43,067,124 | 41,537,948 | 6.23 G | 0.03 | 95.49 | 93.46 | 49.96 |
| CTRL_m_2 | 65,924,404 | 64,026,164 | 9.6 G | 0.03 | 95.41 | 93.34 | 49.72 |
| CTRL_m_3 | 46,657,720 | 45,354,346 | 6.8 G | 0.03 | 95.5 | 92.84 | 50.16 |
| COLD_b_1 | 46,477,660 | 45,445,622 | 6.82 G | 0.03 | 95.55 | 92.86 | 47.14 |
| COLD_b_2 | 44,162,360 | 42,928,266 | 6.44 G | 0.03 | 94.53 | 92.28 | 47.22 |
| COLD_b_3 | 50,962,004 | 49,985,140 | 7.5 G | 0.03 | 95.01 | 91.69 | 46.91 |
| COLD_g_1 | 49,696,118 | 48,158,992 | 7.22 G | 0.03 | 95.41 | 93.37 | 48.65 |
| COLD_g_2 | 40,481,696 | 39,492,698 | 5.92 G | 0.03 | 94.58 | 91.9 | 48.73 |
| COLD_g_3 | 48,323,886 | 46,856,638 | 7.03 G | 0.03 | 95.47 | 93.45 | 48.67 |
| COLD_l_1 | 50,203,248 | 49,048,986 | 7.36 G | 0.03 | 95.81 | 92.83 | 47.83 |
| COLD_l_2 | 48,556,310 | 47,268,912 | 7.09 G | 0.03 | 96.09 | 93.22 | 47.81 |
| COLD_l_3 | 39,395,540 | 38,406,480 | 5.76 G | 0.03 | 95.51 | 93.46 | 48.2 |
| COLD_m_1 | 42,200,762 | 40,583,906 | 6.09 G | 0.03 | 94.65 | 91.97 | 49.28 |
| COLD_m_2 | 41,503,944 | 40,542,418 | 6.08 G | 0.03 | 94.84 | 92.22 | 49.88 |
| COLD_m_3 | 59,504,084 | 57,228,452 | 8.58 G | 0.03 | 95.42 | 93.38 | 49.38 |

**Table 2** Summary of mapping rate

| Sample name | Total reads | Total mapped |
| --- | --- | --- |
| CTRL_b_1 | 45,451,890 | 34,089,898(75.00%) |
| CTRL_b_2 | 41,553,878 | 31,059,286(74.74%) |
| CTRL_b_3 | 49,927,048 | 37,415,478(74.94%) |
| CTRL_g_1 | 44,181,106 | 33,609,504(76.07%) |
| CTRL_g_2 | 44,005,376 | 33,215,122(75.48%) |
| CTRL_g_3 | 55,966,124 | 42,275,830(75.54%) |
| CTRL_l_1 | 55,440,394 | 41,585,680(75.01%) |
| CTRL_l_2 | 62,428,258 | 47,195,966(75.60%) |
| CTRL_l_3 | 54,116,398 | 41,226,728(76.18%) |
| CTRL_m_1 | 41,537,948 | 31,715,050(76.35%) |
| CTRL_m_2 | 64,026,164 | 48,398,060(75.59%) |
| CTRL_m_3 | 45,354,346 | 34,638,152(76.37%) |
| COLD_b_1 | 45,445,622 | 33,938,562(74.68%) |
| COLD_b_2 | 42,928,266 | 31,721,700(73.89%) |
| COLD_b_3 | 49,985,140 | 36,851,524(73.72%) |
| COLD_g_1 | 48,158,992 | 36,595,452(75.99%) |
| COLD_g_2 | 39,492,698 | 29,972,838(75.89%) |
| COLD_g_3 | 46,856,638 | 35,817,364(76.44%) |
| COLD_l_1 | 49,048,986 | 37,676,416(76.81%) |
| COLD_l_2 | 47,268,912 | 36,163,152(76.51%) |
| COLD_l_3 | 38,406,480 | 29,743,184(77.44%) |
| COLD_m_1 | 40,583,906 | 31,233,666(76.96%) |
| COLD_m_2 | 40,542,418 | 30,937,248(76.31%) |
| COLD_m_3 | 57,228,452 | 43,495,246(76.00%) |

**Table 3** Databases information

| Database name | Full name | Web link |
| --- | --- | --- |
| Nr | NCBI non-redundant protein sequences database | <ftp://ftp.ncbi.nih.gov/blast/db> |
| Nt | NCBI nucleotide sequences database | <ftp://ftp.ncbi.nih.gov/blast/db> |
| Pfam | Pfam protein family database | <http://xfam.org/> |
| KOG | EuKaryotic Orthologous Groups | <https://genome.jgi.doe.gov/Tutorial/tutorial/kog.html> |
| Swiss-Prot | Swiss-Prot/UniProtKB | <http://www.uniprot.org/> |
| KEGG | Kyoto Encyclopedia of Genes and Genome | <https://www.genome.jp/kegg/> |
| GO | Gene Ontology | <http://www.geneontology.org/> |

**Table 4** Software information

| Function | Software | Version | Parameters |
| --- | --- | --- | --- |
| Assembly | Trinity | v2.4.0 | min_kmer_cov:1 |
| Clustering analysis | Corset | v1.05 | Default |
| Gene function annotation | diamond | v0.8.22 | NR, Swiss-Prot: e-value = 1e-5, --more-sensitive;  KOG: e-value = 1e-3, --more-sensitive |
|  | NCBI blast 2.2.28+ | v2.2.28+ | e-value = 1e-5 |
|  | KAAS | r140224 | e-value = 1e-10 |
|  | hmmscan | HMMER 3 | e-value = 0.01 |
|  | blast2go | b2g4pipe_v2.5 | e-value = 1.0E-6 |
| Reads mapping and gene quantification | RSEM | v1.2.15 | bowtie2: mismatch 0 |
| Difference expression analysis | DEGSeq | 1.12.0 | padj<0.05 |
|  | DESeq | 1.10.1 | / |
|  | edgeR | 3.0.8 | / |
| GO enrichment | GOSeq，topGO | 1.10.0, 2.10.0 | Corrected P-Value<0.05 |
| KEGG enrichment | KOBAS | v2.0.12 | Corrected P-Value<0.05 |

**Table 5** The primer sequences of genes for qPCR validation

| Gene symbol | Protein/Function | Primer name | Primer sequence (5’-3’) | Product size |
| --- | --- | --- | --- | --- |
| *HSP70* | Heat shock 70 KDa protein | HSP70-2F | TATCTGGGGCAGAAGGTGAC | 245 bp |
|  |  | HSP70-2R | CCGTCTTCAATGGTCAGGAT |  |
| *CRIBP* | Cold inducible RNA binding protein | CRIBP-1F | TGACGAGGGAAAGCTCTTCA | 216 bp |
|  |  | CRIBP-1R | CGGCCATCAACAGACTTTCC |  |
| *TULP4* | Tubby-related protein 4 isoform X1 | 81056-1F | AAAGCGTTAGTCTGAGCCCT | 228 bp |
|  |  | 81056-1R | ACGCTTCATTACACCGATGC |  |
| WWP2 | NEDD4-like E3 ubiquitin-protein ligase WWP2 | 111433-2F | TTCACAGTCTCACGGCAGAT | 250 bp |
|  |  | 111433-2R | TCTAGGTGTCCAGTGCGTTT |  |
| PPARD | Peroxisome proliferator-activated receptor delta | 98981-1F | GAGCACCACCCAACAAAGAG | 244 bp |
|  |  | 98981-1R | TCTCTCGTCACAAAGCCCTT |  |
| *CUL9* | Cullin-9-like isoform X3 | 108996-1F | GTATGCCAGCACCTTTCGAG | 210 bp |
|  |  | 108996-1R | TGCTCAGCCATGGTCAGTAA |  |
| *USP4* | Ubiquitin carboxyl-terminal hydrolase 4 | 92821-1F | TGCTGGTACCCTGAGAACTG | 165 bp |
|  |  | 92821-1R | AGCTGAGATCAACCGGGAAA |  |
| *βACTIN* | Inner Reference | βACTIN-F | TCTTCCAGCCTTCCTTCCTG | 127 bp |
|  |  | βACTIN-R | GTACCTCCAGACAGCACAGT |  |


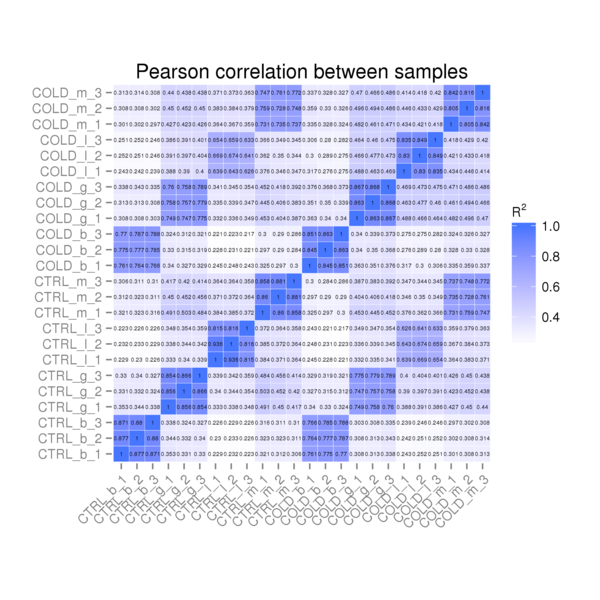


**Fig.1** Heatmap of pearson correlation between samples


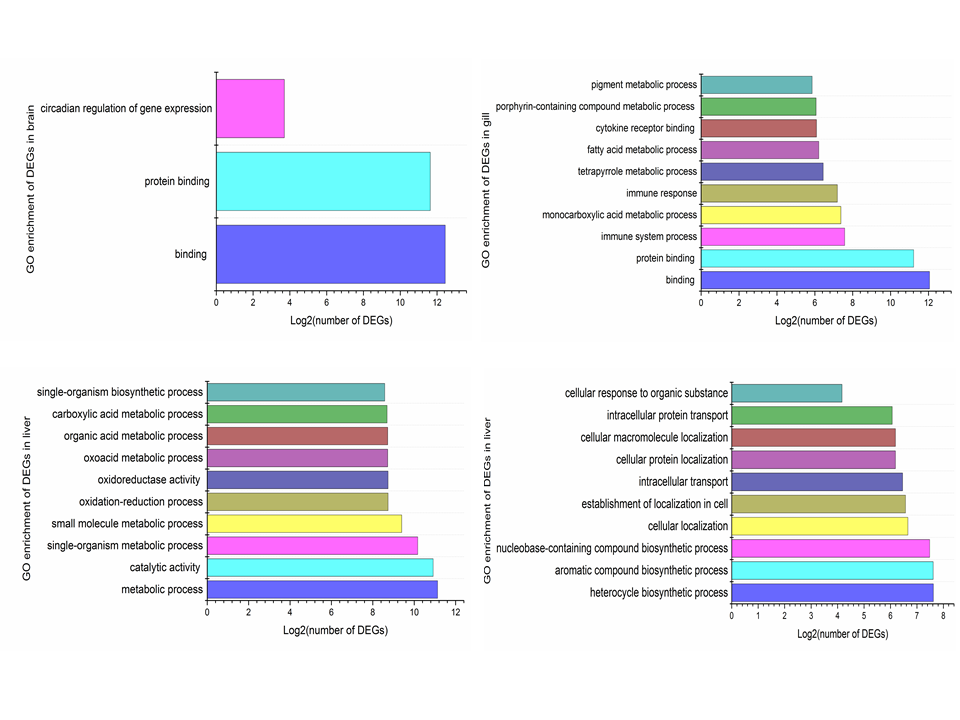


**Fig. 2** Gene Ontology (GO) enrichment of the differently expressed genes (DEGs). Main GO terms of the acute cold induced DEGs enriched from brain, gill, liver and muscle tissues of *Puntius tetrazona*.
